# Altered gravity and time perception: a brief review

**DOI:** 10.3389/fpsyg.2025.1735072

**Published:** 2026-01-20

**Authors:** Amir Jahanian Najafabadi, Carolyn Kroger

**Affiliations:** 1 Department of Cognitive Neuroscience, Bielefeld University, Bielefeld, Germany; 2 Department of Otolaryngology – Head and Neck Surgery, Kresge Hearing Research Institute, University of Michigan, Ann Arbor, MI, United States

**Keywords:** behavioral performance, gravity, perception, spaceflight, temporal judgment, virtual reality

## Abstract

Gravity is fundamental to life on Earth, shaping biological systems and perceptual expectations. Understanding how gravity influences cognition is critical as humans increasingly encounter altered-gravity environments through spaceflight and parabolic flights. This mini-review synthesizes emerging findings on how deviations from Earth-normal gravity affect human time perception. Studies across fields of psychology, neuroscience, and physiology suggest that time perception reflects the dynamic integration of multisensory and bodily signals anchored to Earth's gravitational constraints. We identify key gaps in linking physiological changes to behavioral performance and in extending research to longer time scales, areas needing further advancement to benefit space travelers and offer insights into mechanisms of human timing.

**Review pre-registration:**

PROSPERO CRD42024577286.

## Introduction

Gravity is an ever-present force that has shaped the evolution of physiological and cognitive processes (Adamopoulos et al., 2021; Morey-Holton, 2003). However, humans increasingly encounter environments where gravity conditions differ from Earth's through parabolic flights and space travel, as well as analogous experiences in virtual or augmented reality (VR/AR). These scenarios provide novel interactions with altered gravitational cues, motivating a recent surge in scientific research on the perceptual and biological effects of such alterations. Existing reviews have focused on how Earth's gravity influences aspects of perception and action, such as spatial orientation and motion (Dakin and Rosenberg, 2018; Jörges and López-Moliner, 2017; Lacquaniti et al., 2015; Zago and Lacquaniti, 2005). This review considers recent studies on how Earth-discrepant gravity influences time perception.

Accurate timing is crucial for predicting and coordinating movements in response to environmental stimuli (Breska and Ivry, 2018; Coull and Nobre, 2008; Herbst et al., 2022). Time perception may be distorted under various internal or external conditions, requiring adaptive strategies to achieve task goals. This is of practical consideration for aerospace training and preparation, as astronauts undertake complex tasks in altered-gravity environments. Despite the ubiquity and relevance of time as a feature of human behavior and cognition, much remains unknown about the psychological and neurological mechanisms of time perception, and even less is known about how these processes are affected by gravity. Understanding gravitational effects on time perception may provide insight into the underlying mechanisms of human timing under normal Earth gravity (1 g) conditions, as well as improving the health and performance of astronauts during and after space travel.

The goal of this preregistered review is to clarify patterns of results from empirical studies examining time perception in differing gravitational contexts and how physiological changes may mediate these perceptual effects to identify knowledge gaps and potential targets for future research. In the first section, we focus on recent evidence for gravitational effects on time perception across a variety of timing tasks. We consider the effects of altered gravitational cues on time perception contrasted against the temporal referent of persistent gravitational forces on Earth. Second, we focus on how transient or persistent physiological changes may be linked to altered time perception by reviewing relations between gravitational forces and neural and physiological processes relevant to timing and time perception. Finally, we discuss open questions and future directions for research.

## Altered gravity affects time perception

Gravity affects our perception of time in two key ways: indirectly by influencing the motion of external objects in the world, or directly through changes in physiological or sensory inputs. A substantial body of evidence links the perception of visual object motion through space to time perception (e.g., Brown, 1995; Conway et al., 2016; He et al., 2023; Huang and Jones, 1982; Vidaud-Laperrière et al., 2022), with fewer studies considering the mediating role of gravity on this space-time relation (for a review see: Lacquaniti et al., 2015). From a Bayesian estimation framework, human development and evolution within Earth's gravity provides a strong prior for expected motion physics, and deviations from this prior may induce temporal errors (for review; Jörges and López-Moliner, 2017). Changes in gravitational forces, which are typically stable when at rest on Earth, can also cause physiological symptoms, such as those related to cephalic fluid shifts, affecting the optic and vestibular systems (Hallgren et al., 2016; Lawson et al., 2016; Mader et al., 2013; Norsk et al., 2015). Thus, studying how gravitational forces affect time perception can involve experimentally manipulating the motion kinematics of objects in the visual field or by altering vestibular or proprioceptive feedback. The studies reviewed in this section used various approaches to test time perception under different experimental gravitational environments, including simulated and real gravitational changes experienced by astronauts on the International Space Station (ISS) or during parabolic flight.

### Simulated gravity changes

#### Visual motion kinematics

Gravity affects how objects in the natural world move. Human observers primarily experience visual events in an upright orientation, whereby objects in free-fall accelerate downward. Both the orientation and acceleration of motion have been implicated in timing judgments. Objects moving downward in the visual field produce more accurate duration judgments than objects moving in an orthogonal plane (Moscatelli and Lacquaniti, 2011; Torok et al., 2019). Moscatelli and Lacquaniti (2011) found that participants more accurately judged the duration of an object moving a fixed distance with constant acceleration (9.81 m/s^2^–approximate free-fall acceleration in Earth's gravity) when it was moving downward (congruent with gravity) than upward or sideways. This effect was present even when participants were lying on their side, suggesting that they incorporate proprioceptive gravitational information in addition to visual orientation for duration perception. In a VR study, Torok et al. (2019) found a similar gravitational bias for objects moving a fixed distance under normal and reduced acceleration conditions that simulated Earth and Mars gravity, further supporting the downward orientation-based bias. These studies indicate that, while the visual system may flexibly account for factors affecting acceleration, such as air resistance, duration perception is best for objects moving in a gravity-congruent direction.

Other evidence suggests that gravitational acceleration may be more relevant for the timing of self-motion. One study manipulated visual self-motion on a simulated rollercoaster while participants performed temporal predictions (Indovina et al., 2013). Conditions included downward acceleration or upward deceleration consistent with gravity, and horizontal motion with matched acceleration and deceleration motion parameters (null gravity conditions). Results showed that acceleration and orientation affected the temporal prediction of self-motion. Participants produced anticipatory (early) prediction responses under acceleration and delayed (late) responses under deceleration compared to a constant velocity control condition, and earlier temporal anticipation for downward compared to horizontal acceleration.

Another type of self-motion timing affected by visual kinematics is the temporal control of limb movements during synchronization with a steady (i.e., isochronous) visual rhythm. Previous work has shown that participants exhibit better synchronization performance when tapping a finger with a visual metronome featuring continuous-motion properties (i.e., a bouncing ball) compared to discrete flashes (i.e., a blinking light; Iversen et al., 2015). One study by Gan et al. (2015) showed improved finger-tapping synchronization to a bouncing ball with a realistic acceleration profile matched to Earth's gravity compared to less “natural” motion lacking gravitational acceleration. Thus, gravity-congruent motion enhanced temporal prediction, increasing precision in repetitively timed movements. Another study showed better synchronization with accelerating compared to decelerating motion kinematics, but no orientation effects (Pérez et al., 2023). Overall, altering visual gravitational acceleration and orientation affects temporal prediction during passive and active self-motion tasks.

#### Physiological cues

The simulation studies described above lack physiologically-coherent changes under altered-gravity conditions, producing a mismatch in perceptual and physiological cues. While the visual system is experiencing altered gravity, the observer is still subject to Earth's gravitational inputs to vestibular, proprioceptive, and cardiovascular systems, which, as discussed later in this review, have relevant influences on time perception. Head-down bed rest (HDBR) posture has been used since the 1970s to simulate microgravity (0 g) effects on the body, requiring participants to lie in a −6° inversion for a prolonged period of time (Traon et al., 2007). Vestibular effects of HDBR (Yuan et al., 2018) affect magnitude and time estimation (Gammeri et al., 2023; Qian et al., 2021). Qian et al. (2021) approximated cephalic fluid shifts and vestibular changes experienced in 0 g through an HDBR protocol. Participants performed duration judgments on the 8th and 15th days of consistent HDBR as well as 1 week before and after. Temporal sensitivity thresholds increased from baseline across the HDBR period, returning to baseline 1 week post-HDBR, indicating that physiological changes associated with microgravity contribute to worse time perception. Reduced vestibular inputs due to bilateral vestibulopathy (BVP) share similarities with microgravity effects, wherein fluid-filled peripheral vestibular structures lack reliable information from Earth's gravito-inertial forces during head tilt. Clément et al. (2023) compared astronauts' duration perception during spaceflight to that of BVP patients. Astronauts tended to underestimate a 1-min duration in spaceflight compared to on Earth, demonstrating similar duration perception in 0 g as BVP subjects in 1 g.

### Real gravity changes

Gravitational effects have been observed for duration perception and reaction time for humans experiencing weightlessness (i.e., 0 g) during parabolic flight or spaceflight. Clément (2018) evaluated supra-second duration estimation during repeated bouts of 1, 0, and 1.8 g lasting 25–30 s each during parabolic flight sequences. Passengers were required to estimate the duration of a visual marker while performing a digit-reading task to prevent explicit counting of seconds. Durations were underestimated in 0 and 1.8 g relative to 1 g, however, only 0 g conditions produced statistically-significant underestimations. Navarro-Morales et al. (2023) found a similar result for astronauts, estimating 1-min by counting before, during, and after spending 6–8 months on the ISS. Compared to pre- and post-flight estimations, 1-min was underestimated when free-floating on board the ISS. On average, astronauts in 0 g reported that a minute had passed after a duration 15-s shorter compared to normal 1 g. Another study reported similar temporal underestimation while aboard the ISS compared to pre- and post-flight with durations ranging from 2 to 38 s (Kuldavletova et al., 2023). This study additionally showed a trend of increasing temporal error (greater underestimation) across days 17–164 on the ISS with repeated testing for a dual-task requiring simultaneous digit-reading. For reaction time tasks, participants were slower to press a button following a visual cue in 0 g compared to 1 or 1.8 g during parabolic flight (Clément, 2018) and spaceflight (Kuldavletova et al., 2023). Together, results suggest that time “passes faster” in altered gravity, and increased cognitive load may play a role in duration underestimation and slower reaction times produced by astronauts in spaceflight.

## Neurophysiological mediation of gravitational effects on timing

Of utmost consideration for understanding gravitational effects on time perception are potential neurological and physiological mediators. Increases and decreases in gravitational forces on the body cause physiological changes to cardiovascular, vestibular, ocular, and somatosensory systems (Lee et al., 2020; Mergner and Rosemeier, 1998; Reschke and Clément, 2018b; Sarker et al., 2023). In astronauts exposed to different gravity conditions, changes include reduced heart rate and heart rate variability (Fritsch-Yelle et al., 1985; Migeotte et al., 2003; Otsuka et al., 2016), increased saccade latency (Reschke et al., 2017), and altered pupil activity associated with increased intracranial pressure and spaceflight-associated neuro-ocular syndrome (Lee et al., 2020; Sarker et al., 2023). Such bodily functions have been associated with timing processes; thus, altered gravity may affect time perception mechanisms directly or indirectly through neurophysiological mediators.

### Neurophysiological correlates of time perception in Earth's gravity

Time perception has been linked to physiological metrics such as temperature (Van Maanen et al., 2019), heart rate (Pollatos et al., 2014; Arslanova et al., 2023), pupil dilation (Faber, 2017), and eye movements (Ayres et al., 2021). Greater heart rate variability is associated with more accurate reproduction of temporal intervals, especially for intervals between 2 and 25 s (Pollatos et al., 2014). Furthermore, increased pupil size correlates with higher cognitive load and error monitoring during timing tasks (Eckstein et al., 2017; Warda et al., 2022). Changes in arousal also affect time perception, where higher arousal, marked by increased heart rate, body temperature, and pupil diameter, is associated with faster tempo judgments and duration overestimation (Jakubowski et al., 2015; Wearden and Penton-Voak, 1995).

The autonomic nervous system plays a central role in these effects. According to interoceptive theories (Craig, 2002, 2009), time perception arises from signals sent by afferent autonomic pathways conveying the internal states. Popular models of timing suggest that an internal pacemaker's rate and attentional focus on time passage govern subjective timing (Zakay and Block, 1997; Brown, 2008). Complementary neurophysiological evidence indicated that subjective time perception engages partially distinct neural networks depending on sensory context. Proshina et al. (2024) found that duration reproduction under eyes-open and eyes-closed conditions elicited differential activation patterns across parietal, frontal, and cingulate regions using EEG source localization. While subjective temporal judgments did not vary behaviorally across conditions, oscillatory activity in beta and alpha bands correlated with duration estimates within domain-relevant cortical sites, suggesting that context-dependent recruitment of cortical resources may reflect flexible instantiations of time perception processes. Pollatos et al. (2014) showed that sympathovagal balance and interoceptive processing contribute to temporal accuracy, indicating that cardiac signals play a direct role in interval timing. They found that individuals with higher interoceptive sensitivity demonstrated better synchronization performance specifically for a 2-s interval, suggesting that the influence of bodily signals on timing may depend on the duration being judged. Lacquaniti et al. (2015) proposed that the brain incorporates an internal model of gravitational effects with multisensory signals for temporal and spatial tasks, underscoring the integration of multimodal gravity-related information, such as visual object motion and vestibular inputs, for time perception.

### Gravitational effects on neurophysiology

Spaceflight and artificial gravity studies reveal that altered gravitational loads modulate physiological rhythms interconnected with timing. Microgravity reduces parasympathetic tone and heart rate variability, altering autonomic cardiovascular control and potentially destabilizing interoceptive signals important for timing (Fritsch-Yelle et al., 1985; Migeotte et al., 2003; Otsuka et al., 2016). These physiological parameters often persist upon initial return to Earth's gravity and later reverse, with elevated heart rates commonly observed within 24 h post-flight and persisting for days or weeks before returning to baseline. Simultaneously, vestibular perturbations modulate spatial and temporal perception through parietal-insular cortical pathways sensitive to gravitational cues (Denise et al., 2022; Walsh, 2003). The interaction of gravitational cues with visual and vestibular mechanisms further underscores the sensorimotor grounding of temporal representations. Visual-motor coordination and oculomotor dynamics are also influenced by gravity. For example, pupil and saccadic alterations tied to intracranial pressure changes impact timing-related cognitive load and temporal integration during spaceflight (Lee et al., 2020; Sarker et al., 2023; Reschke and Clément, 2018a). Altogether, gravitational modulation of physiological processes, including autonomic cardiac signals, vestibular input, ocular-motor function, and arousal states, forms a complex interplay that affects the experience of time. Understanding these neuro-psychophysiological signatures offers insights into time perception for future space missions (Craig, 2002, 2009; Fritsch-Yelle et al., 1985; Lacquaniti et al., 2013).

Taken together, these lines of research support the notion that the perception of time is subserved by a complex, distributed network including sensory, vestibular, and parietal-insular cortical regions, with some task-specific engagement. Crucially, vestibular and gravitational inputs appear to modulate these mechanisms, and conditions associated with normal Earth gravity provide a fundamental reference for temporal estimation. Emerging evidence from altered gravity environments, neuroimaging, and electrophysiology suggests that temporal processing is not only symbolically abstract but also deeply tied to bodily states and ecological constraints. Thus, these studies provide an indirect link between gravity-based neurophysiological changes to altered time perception.

## Future directions and conclusion

Much remains unknown regarding the effects of altered gravity on time perception via psychological or neurophysiological changes. Although there is a growing literature on gravitational effects on human physiological and perceptual functions, most studies have examined these effects in isolation, requiring causal inferences between relevant changes. Future studies should directly examine the roles of vestibular, visual, and interoceptive systems in time perception under altered gravity. Multimodal neuroimaging and electrophysiological studies during space missions and analog environments could clarify the underlying neural and physiological mechanisms of timing. Longitudinal monitoring of astronauts before, during, and after flight is essential to characterize adaptive changes in temporal processing and recovery. Including cross-modal timing tasks spanning visual, auditory, and tactile domains will help reveal how gravity-related sensory alterations shape integrated time perception. Further, ecologically valid tasks that mimic activities undertaken by astronauts on spaceflight missions will increase the translational relevance of findings.

Continued development and testing of countermeasures such as immersive VR or physiological feedback protocols will be important from both basic and applied scientific perspectives. Considering studies under real gravitational manipulations, isolating sensory and physiological effects using simulations may help disentangle contributions of different sources of information such as visual dynamics and vestibular inputs. Altered gravity not only causes astronauts to feel the effects of changes in vestibular inputs but also fundamentally changes the nature of how objects move around the visual field. VR/AR studies provide an avenue for testing the effects of visual gravitational changes on time perception in the absence of vestibular alteration. Understanding the connection between gravity-based changes and visual information is crucial not only because of physiological impacts on the visual system (Lee et al., 2020; Reschke et al., 2017; Sarker et al., 2023), but because visual object motion directly modulates time perception for stimuli within and across modalities (Brown, 1995; He et al., 2023).

Future studies should go beyond vestibular and proprioceptive to consider auditory, tactile, and interoceptive signals in gravity-modulated timing. Multisensory tasks can distinguish modality-specific from shared effects. How gravity interacts with affective and cognitive states, such as stress or motivation, also warrants attention. Active timing (timed motor actions) should be contrasted with passive timing (perception without action), as altered gravity may affect motor-perceptual integration differently from perception alone. With growing potential for space missions to extend in duration and distance, the systematic investigation of longer timescales should be undertaken. Most prior work has focused on brief (milliseconds-seconds) or intermediate (seconds-minutes) intervals, leaving hours-to-days durations relatively unexplored. Examining circadian (24-h) and ultradian (less than 24-h) rhythms in altered gravity will further clarify mechanisms governing timing across scales.

Finally, advancing formal computational models should explicitly incorporate gravity as a parameter in timing mechanisms. Such models should account for dynamic re-weighting of multisensory inputs, particularly vestibular, visual, proprioceptive, and interoceptive signals, when reliability changes under different gravitational conditions. Bayesian perceptual inference (Jörges and López-Moliner, 2017, 2020; Allred et al., 2023), Bayesian Prediction Error Minimization (PEM; i.e., “distrusting the present”; Kent et al., 2019), and predictive-processing frameworks (Millidge et al., 2024) offer promising foundations as they can simulate how altered gravity introduces temporal biases by modifying prediction errors and sensory precision estimates. State-space adaptation models (e.g., Ravichandran-Schmidt and Hass, 2024) may further help chart how perceptual timing recalibrates over longer missions and, once astronauts return to Earth, predict trajectories of learning, compensation, and recovery.

In conclusion, growing evidence highlights that altered gravity profoundly influences perceptual, neural, and physiological substrates of human timing. These changes manifest as distortions in subjective timing, variability in temporal accuracy, and recalibration of sensorimotor integration. Understanding how gravity reshapes time perception not only informs fundamental cognitive neuroscience but is critical for astronaut performance and wellbeing during long-duration space missions. Continued interdisciplinary research integrating psychology, neurophysiology, vestibular science, and medicine is vital to unraveling these complex processes and to developing effective countermeasures for the challenges of human activities beyond Earth. Such studies may also extend beyond space exploration, offering translational benefits for clinical neuroscience. Simulations such as VR/AR can be harnessed for rehabilitation protocols that allow patients with motor, vestibular, or perceptual disorders to relearn and practice timing-dependent skills in controlled, adaptive environments. In this way, research on gravity and time perception not only advances our readiness for future spaceflight but also enriches therapeutic strategies to support recovery and functional independence on Earth.

## References

[B1] Adamopoulos K. Koutsouris D. Zaravinos A. Lambrou G. I. (2021). Gravitational influence on human living systems and the evolution of species on earth. Molecules 26:2784. doi: 10.3390/molecules26092784 34066886 PMC8125950

[B2] Allred A. R. Kravets V. G. Ahmed N. Clark T. K. (2023). Modeling orientation perception adaptation to altered gravity environments with memory of past sensorimotor states. Front. Neural Circuits 17:1190582. doi: 10.3389/fncir.2023.1190582 37547052 PMC10399228

[B3] Arslanova I. Kotsaris V. Tsakiris M. (2023). Perceived time expands and contracts within each heartbeat. Curr. Biol. 33, 1389–1395.e4. doi: 10.1016/j.cub.2023.02.034 36905931

[B4] Ayres P. Lee J. Y. Paas F. Van Merriënboer J. J. G. (2021). The validity of physiological measures to identify differences in intrinsic cognitive load. Front. Psychol. 12:702538. doi: 10.3389/fpsyg.2021.702538 34566780 PMC8461231

[B5] Breska A. Ivry R. B. (2018). Double dissociation of single-interval and rhythmic temporal prediction in cerebellar degeneration and Parkinson's disease. Proc. Nat. Acad. Sci. 115, 12283–12288. doi: 10.1073/pnas.1810596115 30425170 PMC6275527

[B6] Brown S. W. (1995). Time, change, and motion: the effects of stimulus movement on temporal perception. Percept. Psychophys. 57, 105–116. doi: 10.3758/BF03211853 7885802

[B7] Brown S. W. (2008). “Time and attention: review of the literature,” in Psychology of Time, ed S. Grondin (Bingley: Emerald Group Publishing Ltd.), 111–138.

[B8] Clément G. (2018). Perception of time in microgravity and hypergravity during parabolic flight. Neuroreport 29, 247–251. doi: 10.1097/WNR.0000000000000923 29112678

[B9] Clément G. Kuldavletova O. Macaulay T. R. Wood S. J. Morales D. C. N. Toupet M. . (2023). Cognitive and balance functions of astronauts after spaceflight are comparable to those of individuals with bilateral vestibulopathy. Front. Neurol. 14:1284029. doi: 10.3389/fneur.2023.1284029 37965165 PMC10641777

[B10] Conway L. G. Repke M. A. Houck S. C. (2016). Psychological spacetime: implications of relativity theory for time perception: implications of relativity theory for time perception. SAGE Open 6. doi: 10.1177/2158244016674511

[B11] Coull J. Nobre A. (2008). Dissociating explicit timing from temporal expectation with fMRI. Curr. Opin. Neurobiol. 18, 137–144. doi: 10.1016/j.conb.2008.07.011 18692573

[B12] Craig A. (2009). Emotional moments across time: a possible neural basis for time perception in the anterior insula. Philos. Trans. R. Soc. B Biol. Sci. 364, 1933–1942. doi: 10.1098/rstb.2009.0008 19487195 PMC2685814

[B13] Craig A. D. (2002). How do you feel? Interoception: the sense of the physiological condition of the body. Nat. Rev. Neurosci. 3, 655–666. doi: 10.1038/nrn894 12154366

[B14] Dakin C. J. Rosenberg A. (2018). Gravity estimation and verticality perception. Handb. Clin. Neurol. 159, 43–59. doi: 10.1016/B978-0-444-63916-5.00003-3 30482332 PMC6295197

[B15] Denise P. Harris L. R. Clément G. (2022). Editorial: role of the vestibular system in the perception of time and space. Front. Integr. Neurosci. 16:1067425. doi: 10.3389/fnint.2022.1067425 36440179 PMC9686397

[B16] Eckstein M. K. Guerra-Carrillo B. Singley A. T. M. Bunge S. A. (2017). Beyond eye gaze: what else can eyetracking reveal about cognition and cognitive development? Dev. Cogn. Neurosci. 25, 69–91. doi: 10.1016/j.dcn.2016.11.001 27908561 PMC6987826

[B17] Faber N. J. (2017). Neuromodulation of pupil diameter and temporal perception. J. Neurosci. 37, 2806–2808. doi: 10.1523/JNEUROSCI.0012-17.2017 28298570 PMC5354328

[B18] Fritsch-Yelle J. M. Charles J. B. Jones M. M. Wood M. L. (1985). Microgravity decreases heart rate and arterial pressure in humans. J. Appl. Physiol. 80, 910–914. doi: 10.1152/jappl.1996.80.3.910 8964756

[B19] Gammeri R. Salatino A. Pyasik M. Cirillo E. Zavattaro C. Serra H. . (2023). Modulation of vestibular input by short-term head-down bed rest affects somatosensory perception: implications for space missions. Front. Neural Circuits, 17:1197278. doi: 10.3389/fncir.2023.1197278 37529715 PMC10390228

[B20] Gan L. Huang Y. Zhou L. Qian C. Wu X. (2015). Synchronization to a bouncing ball with a realistic motion trajectory. Sci. Rep. 5:11974. doi: 10.1038/srep11974 26148810 PMC4493690

[B21] Hallgren E. Kornilova L. Fransen E. Glukhikh D. Moore S. T. Clément G. . (2016). Decreased otolith-mediated vestibular response in 25 astronauts induced by long-duration spaceflight. J. Neurophysiol. 115, 3045–3051. doi: 10.1152/jn.00065.2016 27009158 PMC4922620

[B22] He X. Ke Z. Wu Z. Chen L. Yue Z. (2023). The speed and temporal frequency of visual apparent motion modulate auditory duration perception. Sci. Rep. 13:11281. doi: 10.1038/s41598-023-38183-w 37438383 PMC10338538

[B23] Herbst S. K. Obleser J. Van Wassenhove V. (2022). Implicit versus explicit timing—Separate or shared mechanisms? J. Cogn. Neurosci. 34, 1447–1466. doi: 10.1162/jocn_a_01866 35579985

[B24] Huang Y. L. Jones B. (1982). On the interdependence of temporal and spatial judgments. Percept. Psychophys. 32, 7–14. doi: 10.3758/BF03204862 7133950

[B25] Indovina I. Maffei V. Lacquaniti F. (2013). Anticipating the effects of visual gravity during simulated self-motion: estimates of time-to-passage along vertical and horizontal paths. Exp. Brain Res. 229, 579–586. doi: 10.1007/s00221-013-3620-3 23807477

[B26] Iversen J. R. Patel A. D. Nicodemus B. Emmorey K. (2015). Synchronization to auditory and visual rhythms in hearing and deaf individuals. Cognition 134, 232–244. doi: 10.1016/j.cognition.2014.10.018 25460395 PMC4255154

[B27] Jakubowski K. Halpern A. R. Grierson M. Stewart L. (2015). The effect of exercise-induced arousal on chosen tempi for familiar melodies. Psychon. Bull. Rev. 22, 559–565. doi: 10.3758/s13423-014-0687-1 25056004

[B28] Jörges B. López-Moliner J. (2017). Gravity as a strong prior: implications for perception and action. Front. Hum. Neurosci. 11:203. doi: 10.3389/fnhum.2017.00203 28503140 PMC5408029

[B29] Jörges B. López-Moliner J. (2020). Determining mean and standard deviation of the strong gravity prior through simulations. PLoS ONE 15, e0236732. doi: 10.1371/journal.pone.0236732 32813686 PMC7446919

[B30] Kent L. Van Doorn G. Hohwy J. Klein B. (2019). Bayes, time perception, and relativity: the central role of hopelessness. Conscious. Cogn. 69, 70–80. doi: 10.1016/j.concog.2019.01.012 30711789

[B31] Kuldavletova O. Navarro Morales D. C. Quarck G. Denise P. Clément G. (2023). Spaceflight alters reaction time and duration judgment of astronauts. Front. Physiol. 14:1141078. doi: 10.3389/fphys.2023.1141078 37007995 PMC10063900

[B32] Lacquaniti F. Bosco G. Gravano S. Indovina I. La Scaleia B. Maffei V. . (2015). Gravity in the brain as a reference for space and time perception. Multisens. Res. 28, 397–426. doi: 10.1163/22134808-00002471 26595949

[B33] Lacquaniti F. Bosco G. Indovina I. La Scaleia B. Maffei V. Moscatelli A. . (2013). Visual gravitational motion and the vestibular system in humans. Front. Integr. Neurosci. 7:101. doi: 10.3389/fnint.2013.00101 24421761 PMC3872780

[B34] Lawson B. D. Rupert A. H. McGrath B. J. (2016). The neurovestibular challenges of astronauts and balance patients: some past countermeasures and two alternative approaches to elicitation, assessment and mitigation. Front. Syst. Neurosci. 10:96. doi: 10.3389/fnsys.2016.00096 27920669 PMC5118654

[B35] Lee A. G. Mader T. H. Gibson C. R. Tarver W. Rabiei P. Riascos R. F. . (2020). Spaceflight associated neuro-ocular syndrome (SANS) and the neuro-ophthalmologic effects of microgravity: a review and an update. Npj Microgravity, 6:7. doi: 10.1038/s41526-020-0097-9 32047839 PMC7005826

[B36] Mader T. H. Gibson C. R. Pass A. F. Lee A. G. Killer H. E. Hansen H. . (2013). Optic disc edema in an astronaut after repeat long-duration space flight. J. Neuro-Ophthalmol. 33, 249–255. doi: 10.1097/WNO.0b013e31829b41a6 23851997

[B37] Mergner T. Rosemeier T. (1998). Interaction of vestibular, somatosensory and visual signals for postural control and motion perception under terrestrial and microgravity conditions—a conceptual model. Brain Res. Rev. 28, 118–135. doi: 10.1016/S0165-0173(98)00032-0 9795180

[B38] Migeotte P. Prisk G. K. Paiva M. (2003). Microgravity alters respiratory sinus arrhythmia and short-term heart rate variability in humans. Am. J. Physiol. Heart Circ. Physiol. 284, H1995–H2006. doi: 10.1152/ajpheart.00409.2002 12560205

[B39] Millidge B. Tang M. Osanlouy M. Harper N. S. Bogacz R. (2024). Predictive coding networks for temporal prediction. PLoS Comput. Biol. 20:e1011183. doi: 10.1371/journal.pcbi.1011183 38557984 PMC11008833

[B40] Morey-Holton E. R. (2003). “The impact of gravity on life,” in Evolution on Planet Earth: The Impact of the Physical Environment, eds L. J. Rothschild, and A. M. Lister (Amsterdam: Elsevier), 143–159. doi: 10.1016/B978-012598655-7/50036-7

[B41] Moscatelli A. Lacquaniti F. (2011). The weight of time: gravitational force enhances discrimination of visual motion duration. J. Vis. 11:5. doi: 10.1167/11.4.5 21478379

[B42] Navarro-Morales D. C. N. Kuldavletova O. Quarck G. Denise P. Clément G. (2023). Time perception in astronauts on board the International Space Station. Npj Microgravity, 9:6. doi: 10.1038/s41526-023-00250-x 36658133 PMC9852442

[B43] Norsk P. Asmar A. Damgaard M. Christensen N. J. (2015). Fluid shifts, vasodilatation and ambulatory blood pressure reduction during long duration spaceflight. J. Physiol. 593, 573–584. doi: 10.1113/jphysiol.2014.284869 25774397 PMC4324706

[B44] Otsuka K. Cornelissen G. Furukawa S. Kubo Y. Hayashi M. Shibata K. . (2016). Long-term exposure to space's microgravity alters the time structure of heart rate variability of astronauts. Heliyon 2:e00211. doi: 10.1016/j.heliyon.2016.e00211 28050606 PMC5192238

[B45] Pérez O. Monache S. D. Lacquaniti F. Bosco G. Merchant H. (2023). Rhythmic tapping to a moving beat motion kinematics overrules natural gravity. iScience 26:107543. doi: 10.1016/j.isci.2023.107543 37744410 PMC10517406

[B46] Pollatos O. Yeldesbay A. Pikovsky A. Rosenblum M. (2014). How much time has passed? Ask your heart. Front. Neurorobot. 8:15. doi: 10.3389/fnbot.2014.00015 24782755 PMC3988366

[B47] Proshina E. Mitiureva D. Sysoeva O. (2024). Distinct brain systems are involved in subjective minute estimation with eyes open or closed: EEG source analysis study. Front. Neurosci. 18:1506987. doi: 10.3389/fnins.2024.1506987 39748918 PMC11693652

[B48] Qian Y. Jiang S. Jing X. Shi Y. Qin H. Xin B. . (2021). Effects of 15-Day Head-Down bed rest on emotional time perception. Front. Psychol. 12:732362. doi: 10.3389/fpsyg.2021.732362 35002835 PMC8727352

[B49] Ravichandran-Schmidt P. Hass J. (2024). Testing the state-dependent model of subsecond time perception against experimental evidence. Elife. doi: 10.7554/eLife.94418

[B50] Reschke M. F. Clément G. (2018a). Vestibular and sensorimotor dysfunction during space flight. Curr. Pathobiol. Rep. 6, 177–183. doi: 10.1007/s40139-018-0173-y

[B51] Reschke M. F. Clément G. (2018b). Verbal reports of neurovestibular symptoms in astronauts after short-duration space flight. *Acta Astronaut*. 152, 229–234. doi: 10.1016/j.actaastro.2018.08.028

[B52] Reschke M. F. Kolev O. I. Clément G. (2017). Eye-head coordination in 31 space shuttle astronauts during visual target acquisition. Sci. Rep. 7:14283. doi: 10.1038/s41598-017-14752-8 29079792 PMC5660216

[B53] Sarker P. Ong J. Zaman N. Kamran S. A. Waisberg E. Paladugu P. . (2023). Extended reality quantification of pupil reactivity as a non-invasive assessment for the pathogenesis of spaceflight associated neuro-ocular syndrome: a technology validation study for astronaut health. Life Sci. Space Res. 38, 79–86. doi: 10.1016/j.lssr.2023.06.001 37481311

[B54] Torok A. Gallagher M. Lasbareilles C. Ferrè E. R. (2019). Getting ready for Mars: How the brain perceives new simulated gravitational environments. Q. J. Exp. Psychol. 72, 2342–2349. doi: 10.1177/1747021819839962 30852941

[B55] Traon A. P. Heer M. Narici M. V. Rittweger J. Vernikos J. (2007). From space to Earth: advances in human physiology from 20 years of bed rest studies (1986–2006). Eur. J. Appl. Physiol. 101, 143–194. doi: 10.1007/s00421-007-0474-z 17661073

[B56] Van Maanen L. Van Der Mijn R. Van Beurden M. H. P. H. Roijendijk L. M. M. Kingma B. R. M. Miletić S. . (2019). Core body temperature speeds up temporal processing and choice behavior under deadlines. Sci. Rep. 9:10053. doi: 10.1038/s41598-019-46073-3 31296893 PMC6624282

[B57] Vidaud-Laperrière K. Brunel L. Syssau-Vaccarella A. Charras P. (2022). Exploring spatiotemporal interactions: on the superiority of time over space. Atten. Percep. Psychophys. 84, 2582–2595. doi: 10.3758/s13414-022-02546-8 36229633

[B58] Walsh V. (2003). A theory of magnitude: common cortical metrics of time, space and quantity. Trends Cogn. Sci. 7, 483–488. doi: 10.1016/j.tics.2003.09.002 14585444

[B59] Warda S. Simola J. Terhune D. B. (2022). Pupillometry tracks errors in interval timing. Behav. Neurosci. 136, 495–502. doi: 10.1037/bne0000533 36222640 PMC9552500

[B60] Wearden J. H. Penton-Voak I. S. (1995). Feeling the heat: body temperature and the rate of subjective time, revisited. Q. J. Exp. Psychol. B. 48, 129–141. doi: 10.1080/14640749508401443 7597195

[B61] Yuan P. Koppelmans V. Reuter-Lorenz P. De Dios Y. Gadd N. Wood S . (2018). Vestibular brain changes within 70 days of head-down bed rest. Hum. Brain Mapp. 39, 2753–2763. doi: 10.1002/hbm.24037 29528169 PMC6033666

[B62] Zago M. Lacquaniti F. (2005). Visual perception and interception of falling objects: a review of evidence for an internal model of gravity. J. Neural Eng. 2, S198–S208. doi: 10.1088/1741-2560/2/3/S04 16135884

[B63] Zakay D. Block R. A. (1997). Temporal cognition. Curr. Dir. Psychol. Sci. 6, 12–16. doi: 10.1111/1467-8721.ep11512604

